# Direct and indirect reference intervals of 25-hydroxyvitamin D: it is not a real vitamin D deficiency pandemic

**DOI:** 10.11613/BM.2024.020706

**Published:** 2024-06-15

**Authors:** Juan José Perales-Afán, Diego Aparicio-Pelaz, Sheila López-Triguero, Elena Llorente, Juan José Puente-Lanzarote, Marta Fabre

**Affiliations:** 1 Clinical Biochemistry Department, Lozano Blesa University Hospital, Zaragoza, Spain; 2 Instituto de Investigación Sanitaria de Aragón, Zaragoza, Spain

**Keywords:** prospective studies, retrospective studies, vitamin D, season, immunoassay

## Abstract

**Introduction:**

Many studies report vitamin D (25-OH-D) deficiency, although there is no consensus among scientific societies on cut-offs and reference intervals (RI). The aim of this study is to establish and compare RI for serum 25-OH-D by direct and indirect methods.

**Materials and methods:**

Two studies were performed in Zaragoza (Spain). A retrospective study (N = 7222) between January 2017 and April 2019 was used for RI calculation by indirect method and a prospective study (N = 312) with healthy volunteers recruited in August 2019 and February 2020 for direct method. Seasonal differences were investigated. Measurements were performed on Cobas C8000 (Roche-Diagnostics, Basel, Switzerland) using electrochemiluminescence immunoassay technology.

**Results:**

Reference intervals (2.5-97.5 percentile and corresponding 95% confidence intervals, CIs) were as follows: by indirect method 5.6 ng/mL (5.4 to 5.8) - 57.2 ng/mL (55.2 to 59.8), in winter 5.4 ng/mL (5.2 to 5.7) - 55.7 ng/mL (53.6 to 58.4), while in summer 5.9 ng/mL (5.4 to 6.2) - 59.9 ng/mL (56.3 to 62.9). By direct method 9.0 ng/mL (5.7 to 9.5) - 41.4 ng/mL (37.6 to 48.0), in winter 7.4 ng/mL (3.9 to 8.6) - 34.6 ng/mL (30.6 to 51.5), while in summer 13.3 ng/mL (10.1 to 14.1) - 44.1 ng/mL (38.9 to 66.0). In both methods, RIs were higher in summer. A significant difference was observed in 25-OH-D median values between the two methods (P < 0.001).

**Conclusions:**

Reference interval calculation according to the studied area may be a useful tool to adapt the deficiency cut-offs for 25-OH-D. Our data support 25-OH-D values over 12.0 ng/mL for healthy population as sufficient, therefore current recommendations should be updated. In addition, differences in seasonality should be taken into account.

## Graphical abstract

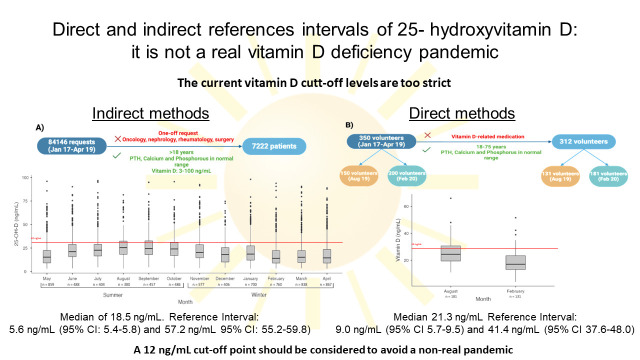

## Highlights

• Addressing vitamin D deficiency, we established reference intervals using direct and indirect methods

• Winter reference intervals (5.4-55.7 ng/mL) contrasts with higher summer values (5.9-59.9 ng/mL), emphasizing the impact of seasons

• Differences between vitamin D reference intervals obtained by direct and indirect methods were detected

• The importance of regional factors is critical; notable distinctions in vitamin D values underscore the need for revised guidelines

## Introduction

Vitamin D is a liposoluble hormone that plays diverse roles in several physiological processes. The vast majority of vitamin D that we use comes from the cutaneous transformation of 7-dehydrocholesterol into cholecalciferol by sunlight. In order to exert its metabolic actions, cholecalciferol requires two enzymatic hydroxylations. The first takes place in the liver, obtaining 25-hydroxyvitamin D (25-OH-D: calcidiol) and the second in the kidney, obtaining the active vitamin D or 1,25-dihydroxyvitamin D (calcitriol). Serum 25-OH-D is the parameter which best reflects the vitamin D status. Furthermore, some factors influence the vitamin D status. Cloud cover, time of day, altitude and air pollution can affect production and show regional variations in vitamin D status (*1*, *2*). Other physiological factors such as gender, age or skin color may also affect the production of vitamin D (*3*). Finally, the absorption and bioavailability of vitamin D is affected by summer/winter variations, malabsorption conditions, medication, sun cream, smoking and obesity (*4*, *5*).

In the last few years, studies link the deficiency of vitamin D to different pathologies such as diabetes, cancer, cardiovascular diseases or infertility (*5*). However, almost none of them has demonstrated the benefit of vitamin D supplementation (*6*, *7*). At the same time, several studies report high prevalence of vitamin D deficiency (*8*). Currently, there is no consensus among the scientific societies about reference intervals (RI) for vitamin D. On one hand, the Institute of Medicine (IOM) specifies that vitamin D deficiency is present when serum concentrations of 25-OH-D are below 20 ng/mL (*9*). Nevertheless, United States Endocrine Society and Japan Endocrine Society consider deficiency defined as 25-OH-D concentrations below 20 ng/mL, and vitamin D insufficiency as 25-OH-D of 21-29 ng/mL (*10*, *11*). On the other hand, Spanish Endocrine Society suggests maintaining serum 25-OH-D concentrations between 30 and 50 ng/mL to achieve health benefits provided by vitamin D (*12*).

The International Federation of Clinical Chemistry and Laboratory Medicine (IFCC) and the Clinical & Laboratory Standard Institute (CLSI) recommend that each clinical laboratory should determine their own RI in cases when RI is not well-established (*13*, *14*). Sometimes, adoption of RIs offered by the diagnostic manufacturer or by external sources could be preferred. Nonetheless, this practice might lead to errors and interpretation problems obtained in the results, especially in analytes as vitamin D, which, as mentioned above, varies cyclically over the year (*1*, *2*, *15*). Direct method is considered as the reference method to obtain RI, but it can be hard to organize, time-consuming and expensive. It is necessary to recruit a large number of well-defined healthy individuals and to implement sample collection, handling and analysis schemes (*16*, *17*). Currently, due to the large volume of routine laboratory data available in the laboratory information system (LIS), indirect methods for RI are becoming more common. These methods are practical and cost-effective, moreover, the sample size of the database is usually adequate after excluding non-compliant samples. However, statistical tools are needed to resolve selection distributions (*17*, *18*).

Accordingly, the present study aims to establish and compare the RI in serum blood for vitamin D by direct and indirect methods in our assistance area in Zaragoza (Spain).

## Materials and methods

### Subjects

The study consisted of establishing 25-OH-D RI using two methodologies: direct and indirect methods. Reference intervals were obtained with the overall data and split by seasonality, according to the months in which the samples were taken. The indirect method patients were distributed in “winter” from November to April and in “summer” from May to October. The direct method patients were already explicitly recruited in February for the “winter” group and August for “summer”.

On one hand, indirect RI methods were obtained from a retrospective study between January 2017 and April 2019 of 25-OH-D requested at the Hospital Clínico Universitario Lozano Blesa (Zaragoza, Spain). Patients with one-off request of 25-OH-D, older than 18 years old and with normal concentrations of parathormone (PTH), calcium and phosphorous were included in the study. Values of 25-OH-D outside the measurement range of the method (3-100 ng/mL) and requests from oncology, nephrology, rheumatology and surgery services were excluded. On the other hand, we conducted a prospective study with volunteers from the Blood and Tissue Bank of Aragón to determine RI using direct methods. Values of 25-OH-D oscillate according to sun exposure therefore samples were collected in periods of highest and lowest sun exposure, August 2019 and February 2020, respectively (*19*). Only volunteers 18-75 years old, with serum PTH, calcium and phosphorus values within the reference intervals were included in the study. Volunteers taking medication that could interfere with vitamin D metabolism were excluded. Through a questionnaire, we ensured that selected volunteers did not exhibit any major illnesses such as diabetes, hypertension or chronic disease, nor any other conditions related to osteocalcic metabolism.

The study was approved by the Research Ethics Committee of the Community of Aragón (C.I. PI19/346) and all patients provided written informed consent.

### Methods

All blood samples were collected using tubes Vacumed with clot activator and separation gel (FL Medical, Torreglia, Italy). Samples were centrifuged at 3500xg for 10 minutes. For indirect method study, all analysis were performed immediately after centrifugation. For direct method study, samples were aliquoted and frozen at - 80 °C until analysis. The samples were carefully thawed and mixed prior to analysis.

Electrochemiluminescence assays were used for 25-OH-D and PTH measurement, whereas calcium and phosphorous were determined by spectrophotometry assays. All analysis were performed by a Cobas C8000 analyzer (Roche Diagnostics, Basel, Switzerland) using their specific reagents. All the method calibrations and quality control assessments were carried out and were within limits throughout all study duration. A comparison of the Elecsys Total Vitamin D III assay was performed using the Center of Disease Control and Prevention (CDC) verification samples with the concentrations assigned by the CDC Vitamin D Reference Laboratory by isotope dilution liquid chromatography-tandem mass spectrometry (ID-LC-MS/MS).

### Statistical analysis

Outliers were identified and eliminated using Reed’s criteria (Reed-Dixon method). Normality of each parameter was tested by using the Kolmogorov-Smirnov test. Normally distributed data are presented as mean and standard deviation, whereas skewed distributed data as median and interquartile range.

Reference intervals were assessed by using statistical procedures recommended by CLSI and IFCC in CLSI/IFCC C28-A3 (*13*). Reference interval was calculated using non-parametric methods, based on the calculation of the 2.5th percentile (p2.5) and 97.5th percentile (p97.5) and their 95% confidence intervals (CI). All statistical analysis were performed using SPSS statistics v22.0 (IBM, Armonk, USA) and Jamovi v2.0 (The jamovi project (2023). jamovi (Version 2.3) (Computer Software)). Differences between parameters with normal distribution were tested using the Student t test, while for skewed distributions the Mann-Whitney U test was used. P values < 0.05 were considered statistically significant.

## Results

### Indirect method

**Figure 1:**
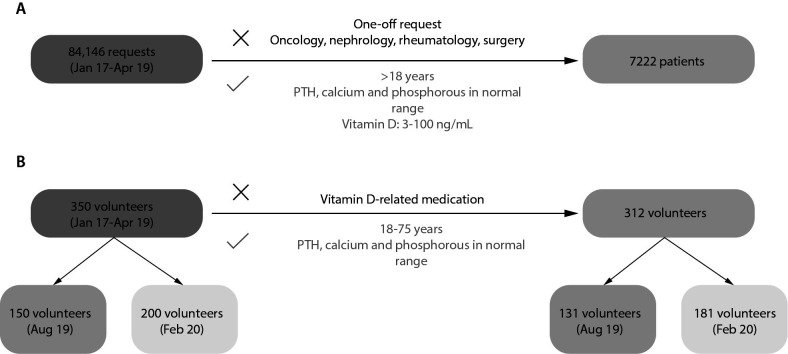
Selection process for calculation of RI by indirect (A) and direct (B) methods, including acceptance and rejection criteria. PTH - parathormone.

A total of 84,146 patient requests for 25-OH-D were extracted from the LIS. After applying the acceptance criteria, 7222 were included and distributed over the course of the months (Figure 1). There were 5248 (73%) female and 1974 (27%) male patients, median age 58 (range 18-99) years. Data distribution was skewed for 25-OH-D results (P < 0.001) and no outliers were detected.

The estimated indirect RI for 25-OH-D was 5.6 ng/mL (95% CI: 5.4 to 5.8) - 57.2 ng/mL (95% CI: 55.2 to 59.8) with a median of 18.5 ng/mL (IQR: 15.5) (Table 1). There was no significant difference in median 25-OH-D values according to sex (male: 17.6 ng/mL (IQR: 34.7), female: 18.9 ng/mL (IQR: 15.5), P = 0.27).

**Table 1:** Study’s reference interval of 25-hydroxyvitamin D defined as percentiles by direct and indirect method

	**Indirect method**				**Direct method**			
	**N**	**Median (IQR)**	**2.5 P (CI)**	**97.5 P (CI)**	**N**	**Median (IQR)**	**2.5 P (CI)**	**97.5 P (CI)**
25-OH-D, ng/mL	7222	18.5 (11.3-26.8)	5.6 (5.4 to 5.8)	57.2 (55.2 to 59.8)	312	21.3 (16.3-27.9)	9.0 (5.7 to 9.5)	41.4 (37.6 to 48.0)
25-OH-D, ng/mL (winter)	4148	16.5 (9.8-24.7)	5.4 (5.2 to 5.7)	55.7 (53.6 to 58.4)	131	17.0 (13.1-23.3)	7.4 (3.9 to 8.6)	34.6 (30.6 to 51.5)
25-OH-D, ng/mL (summer)	3074	21.1 (14.3-28.9)	5.9 (5.4 to 6.2)	59.9 (56.3 to 62.9)	181	24.4 (19.4-30.6)	13.3 (10.1 to 14.1)	44.1 (38.9 to 66.0)
Measurements were performed on Cobas-C8000 (Roche-Diagnostics, Basel, Switzerland) using electrochemiluminescence immunoassay technology. 25-OH-D - 25-hydroxyvitamin D. IQR – interquartile range. 2.5 P – 2.5^th^ percentile. 97.5 P – 97.5^th^ percentile. CI – confidence intervals.								

**Figure 2:**
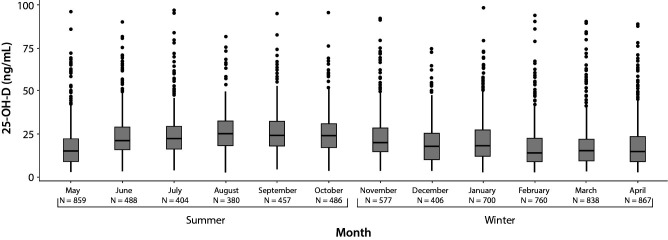
Distribution of vitamin D concentrations by month (indirect method; N = 7222). The boxes represent the interquartile range, the middle horizontal line the median. 25-OH-D - 25-hydroxyvitamin D.

The 7222 patients were distributed in “winter” (N = 4148) and “summer” (N = 3074). Thus, in winter, RI was estimated at 5.4 ng/mL (95% CI: 5.2 to 5.7) - 55.7 ng/mL (95% CI: 53.6 to 58.4) with a median of 16.5 ng/mL (IQR: 9.8-24.7). In summer, RI ranged from 5.9 ng/mL (95% CI: 5.4 to 6.2) - 59.9 ng/mL (95% CI: 56.3 to 62.9) with a median of 21.1 ng/mL (IQR: 14.3-28.9) (Table 1). There were 25-OH-D values significantly higher in summer than in winter (P < 0.001) (Figure 2).

### Direct method

A total of 350 volunteers were recruited and, after criteria selection, 312 volunteers were definitively included. There were 121 (39%) female and 191 (61%) male volunteers, median age 49 (range 18-73) years. Data distribution was skewed for 25-OH-D results (P < 0.001) and no outliers were detected.

Reference interval was estimated at 9.0 ng/mL (95% CI: 5.7 to 9.5) - 41.4 ng/mL (95% CI: 37.6 to 48.0) with a median of 21.3 ng/mL (IQR: 16.3-27.9) (Table 1). There was no significant difference in median 25-OH-D values according to sex (male: 20.3 ng/mL (IQR: 13.0), female: 23.3 ng/mL (IQR: 14.6), P = 0.34).

**Figure 3:**
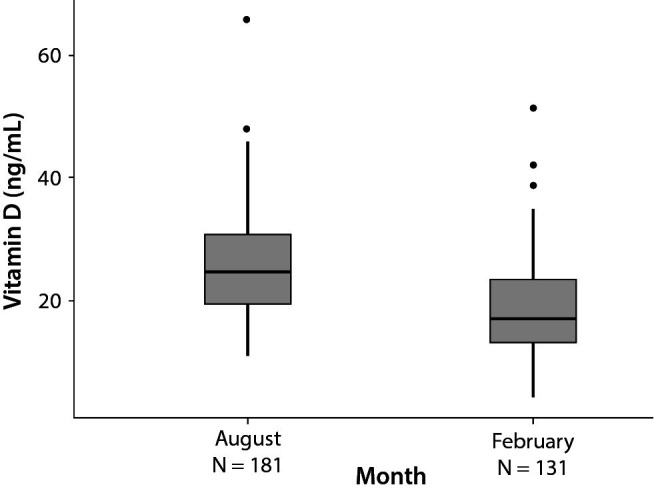
Distribution of vitamin D concentrations by month (direct method; N = 312). The boxes represent the interquartile range, the middle horizontal line the median. 25-OH-D - 25-hydroxyvitamin D.

Volunteers were splitted in “winter” (N = 131) and “summer” groups (N = 181). Winter RI values were 7.4 ng/mL (95% CI: 3.9 to 8.6) - 34.6 ng/mL (95% CI: 30.6 to 51.5) with a median of 17.0 ng/mL (IQR: 13.1-23.3). Summer RI values were 13.3 ng/mL (95% CI: 10.1 to 14.1) - 44.1 ng/mL (95% CI: 38.9 to 66.0) with a median of 24.4 ng/mL (IQR: 19.4-30.6) (Table 1). There was significant difference between the groups (P < 0.05) (Figure 3).

### Comparison between direct and indirect reference intervals

A comparison has been made between the above results for both methods, overall and by seasonality. A significant difference was observed in 25-OH-D median values between the methods (indirect method: 18.5 ng/mL (IQR: 11.3-26.8), direct method: 21.4 ng/mL (IQR: 16.3-27.9); P < 0.001). Focus on the seasonality, summer season data showed a significant difference in the median (indirect method: 21.1 ng/mL (IQR: 14.3-28.9), direct method: 24.4 ng/mL (IQR: 19.4-30.6); P < 0.001). Nevertheless, there was no significant difference in winter season (indirect method: 16.5 ng/mL, direct method: 17.0 ng/mL; P = 0.26).

## Discussion

Our study is the first that establishes RIs by indirect and direct methods for serum 25-OH-D by electrochemiluminescent immunoassay (ECLIA). It shows RI broader by indirect methods than direct methods (5.6-57.2 ng/mL and 9.0-41.4 ng/mL, respectively). This could be explained by the fact that in the direct method we have recruited patients in two specific months, whereas in the indirect method patients were recruited during a long continuous period. In addition, patients with possible pathologies or situations which could fluctuate 25-(OH)-D concentrations have been included in the indirect method calculation. These variables have been controlled in the direct method; consequently, the use by the clinical laboratory specialists of these methods for RI calculations are more suitable. However, RIs and medians for the two methods are similar, hence they are consistent to define 25-OH-D lower RI of our population as insufficient according to most clinical guidelines, which consider a cut-off point of 20 ng/mL (*9*-*12*).

In addition, as vitamin D is directly dependent on UVB radiation and varies seasonally, we checked whether there were significant differences between winter and summer RIs (*20*). We found that 25-OH-D medians are significantly higher in summer than winter, by direct and indirect methods. These results agree with the literature and may be due to the differences in exposure to solar radiation (*21*). In summer, we find the highest atmospheric UV penetration, maximizing the 25-OH-D skin production (*22*). However, practically no skin vitamin D is produced during some winter months due to the low UV penetration, shortening of day length and the greater confinement of people due the bad weather (*19*, *22*). This variation is clearly represented in our results (Figure 2 and 3). It has been shown that vitamin D supplementation can increase 25-OH-D concentrations, but does not completely compensate for seasonal variability (*23*). This seasonal variation must be taken into account to avoid outcomes in the diagnosis of vitamin D deficiency, since values at the limit in summer may be deficient in winter (*20*). Moreover, there is a direct relationship between the highest blood concentrations of 25-OH-D and living near the equator (*24*). In Spain, the latitude is close to 41°, thus 25-OH-D concentrations may vary due to the difference in UV penetration with respect to other locations. The above facts reflect the importance of knowing the origin of the data for RIs calculations.

Vitamin D has become an ordinary measurement to investigate several pathologies in the last years. This fact has meant an increase in number of serum 25-OH-D measurements in clinical laboratories and an overdiagnosis for hypovitamin D in apparently healthy population. In addition, it can be confusing data and information overload for extra-skeletal diseases (*25*). Up to now, cut-off concentrations of 25-OH-D have been published by scientific societies (*21*, *26*, *27*). It should be noted that cut-off values are not obtained according to the evidence-based principle and most societies follow a single point of origin when defining recommended vitamin D concentrations, resulting in a high number of vitamin D-deficient individuals (*5*).

Some studies in Europe have determined RIs by direct methods (11.4-54.4 mg/dL) and by indirect methods (4.8-63.6 mg/dL) (*26*). Other non-European studies, such as Miyamoto *et al.*, concluded by direct method that RI for 25-OH-D in healthy individuals was between 6-29 ng/mL (*21*). These reports show RIs calculated by liquid chromatography-tandem mass spectrometry (LC-MS/MS) instead of ECLIA, which could explain the differences with our RIs. Nonetheless, they are congruent with our results as they calculated a lower RI of less than 20 ng/mL, which relies under our sufficiency limit.

Several studies have investigated vitamin D deficiency worldwide (*8*, *27*). All agree on the existence of vitamin D hypovitaminosis, even considering it a pandemic. Nevertheless, others authors suggest that high prevalence of vitamin D deficiency in healthy populations is artificially created by an unjustifiably high cut-off values of serum 25-OH-D (*27*-*29*). In this regard, as already recommended by some literature, the current cut-off point on deficiency or insufficiency of serum 25-OH-D concentrations should be updated to 12.0 ng/mL (*27*, *28*). This change would be in accordance with our data, which, although still lower, show a trend closer to this new cut-off point. If this cut-off point were updated, cases of vitamin D insufficiency would decrease and probably no longer consider as a pandemic. Supporting our point people with dark skin have 30-40% lower serum vitamin D concentrations than Caucasians, but have equal or higher bone mineral density and lower risk of fractures (*26*). Other authors go further and state that, due to its wide variability, it is inappropriate to use a fixed RI for 25-OH-D in serum. They propose an equation that includes UVB, ethnicity, body mass index, age, sex, and vitamin D supplementation dose (*20*). It remains to be seen whether this idea will prevail.

Several limitations of this study should be noted. Firstly, absence of disease or vitamin D therapy is not guaranteed in the subject population using indirect methods. However, we tried to solve these facts by using specific inclusion criteria (calcium, phosphorus and normal parathormone concentrations or requests for selected services). Secondly, by direct methods we investigated only two specific months. Theoretically, these months represent the extremes in terms of vitamin D values (*19*). Nevertheless, the variations produced in the intermediate months were not been taken into account. It would be interesting to examine these variations in a complete annual study. Thirdly, other facts such as nutritional status, dietary reference intake or ethnic were not considered. Finally, the reference method for the measurement of 25-OH-D is liquid chromatography-mass spectrometry (LC-MS) and we performed it by ECLIA (*5*, *30*). Vitamin D External Quality Assessment Scheme (DEQAS) review shows that, except for LC-MS, the bias for the majority of the currently used instrumentation is still high (*20*).

In conclusion, this study provides evidence about references intervals for serum 25-OH-D by direct and indirect methodologies. According to our data, the current guidelines are too strict and the 12.0 ng/mL cut-off point should be considered to avoid a non-real pandemic.

## Data Availability

The data generated and analyzed in the presented study are available from the corresponding author on request.

## References

[1] O’Sullivan F Laird E Kelly D van Geffen J van Weele M McNulty H Ambient UVB Dose and Sun Enjoyment Are Important Predictors of Vitamin D Status in an Older Population. J Nutr. 2017;147:858–68. 10.3945/jn.116.244079 28331054

[2] Bouillon RA Auwerx JH Lissens WD Pelemans WK. Vitamin D status in the elderly: seasonal substrate deficiency causes 1,25-dihydroxycholecalciferol deficiency. Am J Clin Nutr. 1987;45:755–63. 10.1093/ajcn/45.4.755 3494392

[3] Holick MF Chen TC Lu Z Sauter E. Vitamin D and skin physiology: a D-lightful story. J Bone Miner Res. 2007;22:V28–33. 10.1359/jbmr.07s211 18290718

[4] Gonzalez-Chica D Stocks N. Changes to the frequency and appropriateness of vitamin D testing after the introduction of new Medicare criteria for rebates in Australian general practice: evidence from 1.5 million patients in the NPS Medicine Insight database. BMJ Open. 2019;9:e024797. 10.1136/bmjopen-2018-024797 30852539 PMC6429877

[5] Nikolac Gabaj N Unic A Miler M Pavicic T Culej J Bolanca I In sickness and in health: pivotal role of vitamin D. Biochem Med (Zagreb). 2020;30:020501. 10.11613/BM.2020.020501 32550812 PMC7271749

[6] Pittas AG Dawson-Hughes B Sheehan P Ware JH Knowler WC Aroda VR Vitamin D Supplementation and Prevention of Type 2 Diabetes. N Engl J Med. 2019;381:520–30. 10.1056/NEJMoa1900906 31173679 PMC6993875

[7] Dahma G Reddy G Craina M Dumitru C Popescu A Stelea L The Effects of Vitamin D Supplementation before 20 Weeks of Gestation on Preeclampsia: A Systematic Review. J Pers Med. 2023;13:996. 10.3390/jpm13060996 37373985 PMC10300879

[8] Siddiqee MH Bhattacharjee B Siddiqi UR. MeshbahurRahman M. High prevalence of vitamin D deficiency among the South Asian adults: a systematic review and meta-analysis. BMC Public Health. 2021;21:1823. 10.1186/s12889-021-11888-1 34627207 PMC8501935

[9] Ross AC Manson JE Abrams SA Aloia JF Brannon PM Clinton SK The 2011 report on dietary reference intakes for calcium and vitamin D from the Institute of Medicine: what clinicians need to know. J Clin Endocrinol Metab. 2011;96:53–8. 10.1210/jc.2010-2704 21118827 PMC3046611

[10] Holick MF Binkley NC Bischoff-Ferrari HA Gordon CM Hanley DA Heaney RP Evaluation, Treatment, and Prevention of Vitamin D Deficiency: an Endocrine Society Clinical Practice Guideline. J Clin Endocrinol Metab. 2011;96:1911–30. 10.1210/jc.2011-0385 21646368

[11] Okazaki R Ozono K Fukumoto S Inoue D Yamauchi M Minagawa M Assessment criteria for vitamin D deficiency/insufficiency in Japan: proposal by an expert panel supported by the Research Program of Intractable Diseases, Ministry of Health, Labour and Welfare, Japan, the Japanese Society for Bone and Mineral Research and the Japan Endocrine Society (Opinion). J Bone Miner Metab. 2017;35:1–5. 10.1007/s00774-016-0805-4 27882481

[12] Varsavsky M Rozas Moreno P Becerra Fernández A Luque Fernández I Quesada Gómez JM Ávila Rubio V Recommended vitamin D levels in the general population. Endocrinol Diabetes Nutr. 2017;64:7–14. 10.1016/j.endinu.2016.11.002 28440763

[13] Clinical and Laboratory Standards Institute (CLSI). Defining, Establishing, and Verifying Reference Intervals in the Clinical Laboratory; Approved Guideline -Third Edition. CLSI Document C28-A3. Wayne, PA: CLSI; 2008.

[14] Solberg HE. International Federation of Clinical Chemistry (IFCC), Scientific Committee, Clinical Section, Expert Panel on Theory of Reference Values, and International Committee for Standardization in Haematology (ICSH), Standing Committee on Reference Values. Approved Recommendation (1986) on the theory of reference values. Part 1. The concept of reference values. J Clin Chem Clin Biochem. 1987;25:337–42. 10.1016/0009-8981(87)90224-5 3612033

[15] Zeljkovic A Csuzdi Balog Z Dukai E Vekic J Jelic-Ivanovic Z Spasojevic-Kalimanovska V. Indirect reference intervals for haematological parameters in capillary blood of pre-school children. Biochem Med (Zagreb). 2021;31:010709. 10.11613/BM.2021.010709 33594298 PMC7852301

[16] Shaw JL Binesh Marvasti T Colantonio D Adeli K. Pediatric reference intervals: challenges and recent initiatives. Crit Rev Clin Lab Sci. 2013;50:37–50. 10.3109/10408363.2013.786673 23656169

[17] Ozarda Y Ichihara K Jones G Streichert T Ahmadian R. Comparison of reference intervals derived by direct and indirect methods based on compatible datasets obtained in Turkey. Clin Chim Acta. 2021;520:186–95. 10.1016/j.cca.2021.05.030 34081933

[18] Haeckel R Wosniok W Streichert T Members of the Section Guide Limits of the DGKL. Review of potentials and limitations of indirect approaches for estimating reference limits/intervals of quantitative procedures in laboratory medicine. J Lab Med. 2021;45:35–53. 10.1515/labmed-2020-0131

[19] Reusch J Ackermann H Badenhoop K. Cyclic changes of vitamin D and PTH are primarily regulated by solar radiation: 5-year analysis of a German (50 degrees N) population. Horm Metab Res. 2009;41:402–7. 10.1055/s-0028-1128131 19241329

[20] Ferrari D Lombardi G Banfi G. Concerning the vitamin D reference range: pre-analytical and analytical variability of vitamin D measurement. Biochem Med (Zagreb). 2017;27:030501. 10.11613/BM.2017.030501 28900363 PMC5575654

[21] Miyamoto H Kawakami D Hanafusa N Nakanishi T Miyasaka M Furutani Y Determination of a Serum 25-Hydroxyvitamin D Reference Ranges in Japanese Adults Using Fully Automated Liquid Chromatography-Tandem Mass Spectrometry. J Nutr. 2023;153:1253–64. 10.1016/j.tjnut.2023.01.036 36806449

[22] Raymond-Lezman JR Riskin SI. Benefits and Risks of Sun Exposure to Maintain Adequate Vitamin D Levels. Cureus. 2023;15:e38578. 10.7759/cureus.38578 37284402 PMC10239563

[23] Won JW Jung SK Jung IA Lee Y. Seasonal Changes in Vitamin D Levels of Healthy Children in Mid-Latitude, Asian Urban Area. Pediatr Gastroenterol Hepatol Nutr. 2021;24:207–17. 10.5223/pghn.2021.24.2.207 33833976 PMC8007836

[24] Wacker M Holick MF. Sunlight and Vitamin D: A global perspective for health. Dermatoendocrinol. 2013;5:51–108. 10.4161/derm.24494 24494042 PMC3897598

[25] Aralica M Šupak Smolčić V Turk Wensveen T Hrabrić Vlah S Selar M Bilić Zulle L. An analysis of the vitamin D overtesting in a tertiary healthcare centre. Biochem Med (Zagreb). 2022;32:020701. 10.11613/BM.2022.020701 35464748 PMC8996321

[26] Alonso N Zelzer S Eibinger G Herrmann M Vitamin D. Metabolites: Analytical Challenges and Clinical Relevance. Calcif Tissue Int. 2023;112:158–77. 10.1007/s00223-022-00961-5 35238975 PMC8892115

[27] Cashman KD Dowling KG Škrabáková Z Gonzalez-Gross M Valtueña J De Henauw S Vitamin D deficiency in Europe: pandemic? Am J Clin Nutr. 2016;103:1033–44. 10.3945/ajcn.115.120873 26864360 PMC5527850

[28] Shah D Gupta P Vitamin D. Deficiency: Is The Pandemic for Real? Indian J Community Med. 2015;40:215–7. 10.4103/0970-0218.164378 26435592 PMC4581139

[29] Manson JE Brannon PM Rosen CJ Taylor CL Vitamin D. Deficiency - Is There Really a Pandemic? N Engl J Med. 2016;375:1817–20. 10.1056/NEJMp1608005 27959647

[30] Carter GD Berry J Durazo-Arvizu R Gunter E Jones G Jones J Hydroxyvitamin D assays: An historical perspective from DEQAS. J Steroid Biochem Mol Biol. 2018;177:30–5. 10.1016/j.jsbmb.2017.07.018 28734989

